# Wear Behavior of Austenitic Stainless Steel 316L Plates Fabricated by Wire Arc Additive Manufacturing

**DOI:** 10.3390/ma19153236

**Published:** 2026-07-30

**Authors:** Hussam H. Noor, Mohammed T. Alamoudi, Khalid Alqosaibi, Saleh Alzughaibi, Youssef Alammari, Abdulrahman Alrumayh, Faisal J. Alzahrani

**Affiliations:** 1 Department of Mechanical Engineering, College of Engineering, Taibah University, Medina 42353, Saudi Arabia; hhnoor@taibahu.edu.sa; 2 Advanced Materials Technologies Institute, Energy and Industry Sector, King Abdulaziz City for Science and Technology, Riyadh 11442, Saudi Arabia; malamoudi@kacst.gov.sa; 3 Department of Mechanical Engineering, College of Engineering, King Saud University, Riyadh 12372, Saudi Arabia; kalqosaibi@ksu.edu.sa; 4 Department of Mechanical Engineering, College of Engineering, Qassim University, Buraidah 51452, Saudi Arabia; y.alammari@qu.edu.sa (Y.A.); a.alrumayh@qu.edu.sa (A.A.); 5 Department of Mechanical Engineering, Faculty of Engineering-Rabigh, King Abdulaziz University, Jeddah 21589, Saudi Arabia; fjsalzahrani@kau.edu.sa

**Keywords:** WAAM, austenitic stainless steel, 316L, wear, tribology, pin-on-disk, microstructure

## Abstract

**Highlights:**

- WAAM 316L shows stable COF (~0.6) with speed-dependent fluctuations.
- Wear volume decreases with speed due to the mechanism transition effect.
- Ferrite–austenite microstructure governs tribological performance.
- WAAM thermal history drives grain structure and residual ferrite.

**Abstract:**

Additive manufacturing (AM) of stainless steel has been gaining industry attention in recent years due to the need to manufacture complex steel components. Many sectors stand to benefit from the design flexibility, customization, and rapid production capabilities of AM. However, the industry’s adoption of this technology remains limited due to concerns about the mechanical integrity and reliability of AM products. This experimental study examines the wear and tribological behavior of Wire Arc Additive Manufactured (WAAM) austenitic stainless steel 316L. Pin-on-disk tests were conducted using a 5 mm tungsten carbide ball under dry sliding conditions at normal loads of 1.5 and 2.5 N and sliding speeds between 0.03 and 0.229 m/s. The results showed that the coefficient of friction remained relatively stable at approximately 0.6, while wear volume generally decreased with increasing sliding speed. Lower normal loads resulted in lower wear volume, whereas the wear factor showed only limited sensitivity to the applied load. Optical microscopy revealed a ferrite–austenite microstructure with residual δ-ferrite that contributes to the observed wear behavior. These findings demonstrate the suitability of WAAM-produced 316L stainless steel for tribological applications requiring stable frictional performance.

## 1. Introduction

Developing novel manufacturing processes and optimizing existing ones have been within the scope of many researchers. Additive manufacturing (AM) is one of the novel manufacturing processes that emerged in the 1980s [1]. This paper focuses on one of these seven categories, namely Direct Energy Deposition (DED). This AM process uses an energy source to melt deposited metal that follows a predetermined path to produce a three-dimensional desired object [2]. DED manufacturing processes are commonly classified based on two criteria: the energy source or the type of feedstock [3,4]. This paper focuses on Wire Arc Additive Manufacturing (WAAM), a DED-based technique that employs an electric arc as the energy source and wire as the feedstock material. WAAM utilizes an arc as the heat source for melting a metal wire spool deposited onto the building platform to manufacture the desired object [5]. WAAM’s reliance on wire feedstock and arc welding technology provides an efficient alternative to powder-based AM methods, enabling layer-by-layer deposition with minimal waste [6]. WAAM advancements, such as real-time monitoring and process optimization, have expanded its applicability in manufacturing [7]. The WAAM process is preferred over other AM processes because it has low equipment cost, minimum material waste, better interlayer adhesion strength, high production rate, and low product cost [8]. However, this manufacturing technology has a few setbacks, including residual stresses, surface roughness, heat-related deformation, and stair-stepped surfaces [9].

Various spooled wires made from different metal alloys are used as feedstock for WAAM. Those alloys include titanium, aluminum, nickel, stainless steel, and other materials [10]. Among these materials, stainless steel 316L is an austenitic stainless steel known for its corrosion resistance and strength and is widely used in WAAM applications where durability is essential [11]. This alloy has many applications, including medical and pharmaceutical, marine, chemical and petrochemical, aerospace and automotive, etc. [12,13,14]. SS 316L has superior mechanical properties and is highly ductile and corrosion-resistant [15]. It has been shown that WAAM produces products with good mechanical properties and microstructure using SS 316L [15,16]. However, the thermal cycling involved in WAAM creates unique microstructural characteristics in 316L, which can impact its mechanical and tribological behavior. Recent studies on additively manufactured 316L, especially those using techniques like Selective Laser Melting (SLM), have shown that properties such as layer orientation, surface roughness, and residual stress significantly influence the alloy’s performance [17]. The anisotropic behavior observed in WAAM parts emphasizes the need to understand how WAAM affects wear and fatigue resistance, particularly for demanding applications [18].

Tribology, which focuses on wear, friction, and lubrication, is crucial for evaluating the performance and durability of AM components. Research on AM metals, including high-carbon martensitic stainless steel, demonstrates that AM processing conditions impact tribological behavior and wear mechanisms [19,20]. Studies of SLM-fabricated 316L stainless steel highlight that surface roughness and build direction can significantly affect wear resistance under different sliding and load conditions [21,22]. Investigations into WAAM-fabricated stainless steels such as SS308L and SS347 reveal that process parameters, including heat input and cooling rates, are vital to their tribological performance under dry and elevated temperature conditions [23,24].

WAAM can produce components with distinctive microstructures and graded properties compared to other AM techniques due to its unique thermal profile. For example, SLM-produced 316L stainless steel has been found to exhibit higher wear resistance due to finer microstructures. In contrast, WAAM-fabricated parts may display gradient properties along the build direction, influencing hardness and wear [25]. The dry sliding wear performance of WAAM-manufactured 308 stainless steel has shown varied results based on load and treatment time, resulting in different wear mechanisms, such as abrasive and fatigue delamination wear [26]. This highlights the importance of optimizing WAAM parameters to improve wear resistance for practical applications [27].

In an experimental study, Afshari et al. [28] investigated the heat treatment effect on the microstructure and tribological performance of PH 13-8Mo stainless steel fabricated using WAAM technology. They found that post-printing heat treatment at 500 °C eliminated the delta-ferrite phase from the sample, resulting in an isotropic scratch and wear response. They concluded that WAAM-produced parts can better substitute wrought counterparts for wear-resistance applications. In another study [29], the researchers investigated the tribology and wear behavior of stainless steel 316L samples made with Selective Laser Melting (SLM). Their investigation analyzed the wear behavior of the samples at different temperatures. The results indicated that at 300 °C and 400 °C, the SLM samples had a twofold less wear track than the conventional samples. They pointed out that the SLM samples may be a better alternative for high-temperature wear-resistance applications. Vineesh et al. [30] investigated the dry sliding wear behavior of Direct Metal Laser Sintering (DMLS) samples made of stainless steel 316L. Their investigation focused on high-temperature sliding up to 400 °C. They found that increasing the test temperature reduced the wear resistance due to the oxide glaze appearing on the surface. They also found that the measured wear rates were twofold lower than those of conventional samples. They concluded their analysis by emphasizing that DMLS samples have lower wear rates due to the fine microstructure and the higher hardness than conventional samples. Tanwar and Jhavar [31] conducted a study on the dry sliding wear behavior of WAAM-fabricated structures made of bimetallic 316L and 309 stainless steels. They performed pin-on-disk wear testing and were interested in the tribological behavior of the microstructure, ferrite content, hardness, oxide formation, and the wear surface morphology. Their results showed that the 309 stainless steel had improved wear resistance compared to 316L. They concluded that microstructure tailoring, especially through ferrite formation and hardness enhancement, can significantly improve the wear performance of WAAM-fabricated austenitic stainless steel structures. Alam et al. [32] investigated the influence of build height on the microstructure of WAAM-fabricated 316L stainless steel. They reported finer microstructure and higher hardness for the bottom region of the specimen compared to the upper region. The authors attributed the gradients produced to the faster cooling rate of the bottom layers, leading to a significant impact on the wear performance of WAAM-fabricated 316L stainless steel components.

Although previous studies [27,33,34] have investigated the microstructure, mechanical properties, and corrosion behavior of WAAM-fabricated 316L stainless steel, its tribological response under dry sliding conditions remains less comprehensively addressed. More precisely, limited attention [35,36] has been given to the combined impact of the applied load and the sliding speed on the coefficient of friction, wear volume, and the wear track morphology of WAAM-fabricated 316L stainless steel. This represents an important knowledge gap because WAAM products may be involved in systems undergoing sliding contact, frictional stability, and material loss. This research provides a controlled screening-level tribological assessment of WAAM-fabricated stainless steel. The contribution of this study lies in establishing a comparative understanding of the wear response of WAAM-fabricated 316L stainless steel. At the same time, direct application-level validation requires testing under representative service conditions.

## 2. Materials and Methods

### 2.1. WAAM Specimen Preparation

**Figure 1:**
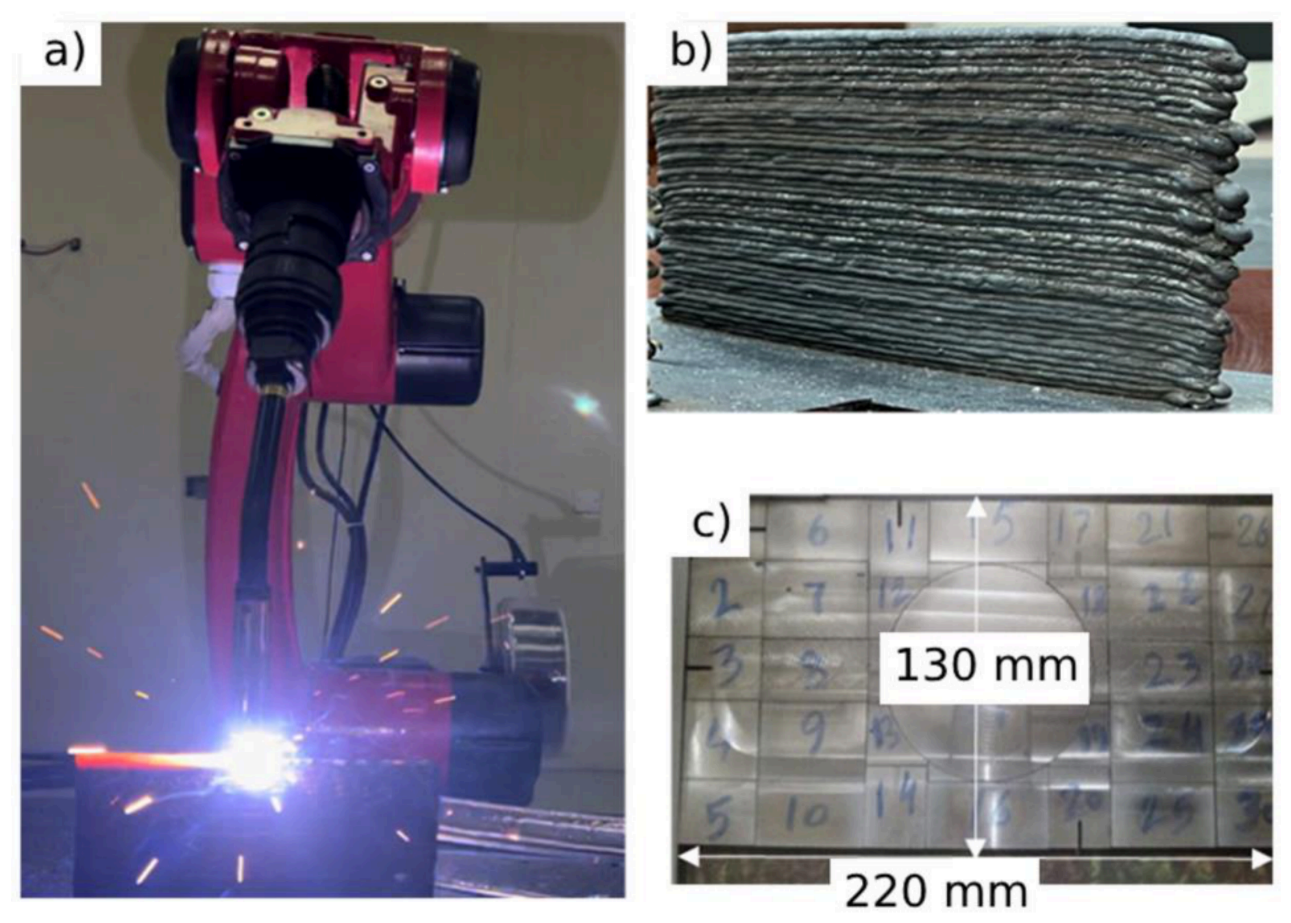
(**a**) Borunte 6-axis robot with an Ehave CM350 welding machine; (**b**) the printed plate via WAAM technology showing the as-deposited sample; (**c**) the printed plate after machining and labeled for cutting multiple samples for investigations.

The workpiece used is made of stainless steel 316L. The samples were fabricated via WAAM technology. WAAM was facilitated using a Borunte 6-axis robot (Borunte Robot Co., Ltd., Dongguan, China) paired with an Ehave CM350 welding machine (Ehave Technology Co., Ltd., Guangzhou, China), shown in Figure 1a, in the Additive Manufacturing Laboratory at Qassim University, Buraidah, Saudi Arabia. Austenitic stainless steel 316L wire with a diameter of 0.8 mm was used as the feedstock material, while a steel substrate served as the build platform. A rectangular plate measuring 220 mm in length and 150 mm in height was printed using WAAM technology, with the printing direction oriented vertically. The voltage was set at E = 20 V, the current was I = 140 A, and ν_w = 3 mm/s. The shielding gas was argon at 99% purity. The printed plate is shown in Figure 1b.

Subsequently, the printed plate underwent CNC machining in a local shop and was cut into the specified shape using a wire cutter to prepare it for testing, as shown in Figure 1c. The samples were then prepared for microstructure, tribology, and hardness analysis. The circular sample at the center was ready for a rotating disk wear test. Display quotations of over 40 words, or as needed.

### 2.2. Metallographic Sample Preparation

The microstructure of the multilayered structure was analyzed at the midsection of the structure’s face. Specimens for metallographic analysis and mechanical testing were prepared using standard metallography techniques, starting with sectioning the samples using a high-speed precision diamond saw. The cut specimens were ground sequentially with abrasive papers of increasing fineness, followed by polishing with 1-micron and 0.5-micron alumina suspensions to achieve a mirror-like finish. The samples were then etched for 45 s in a solution of hydrochloric acid (HCl) and nitric acid (HNO_3_) mixed in a 3:1 ratio to reveal the microstructure. An Olympus optical microscope (Olympus Corporation, Shinjuku, Japan) was employed for the microstructural examination.

### 2.3. Wear Testing Setup

**Figure 2:**
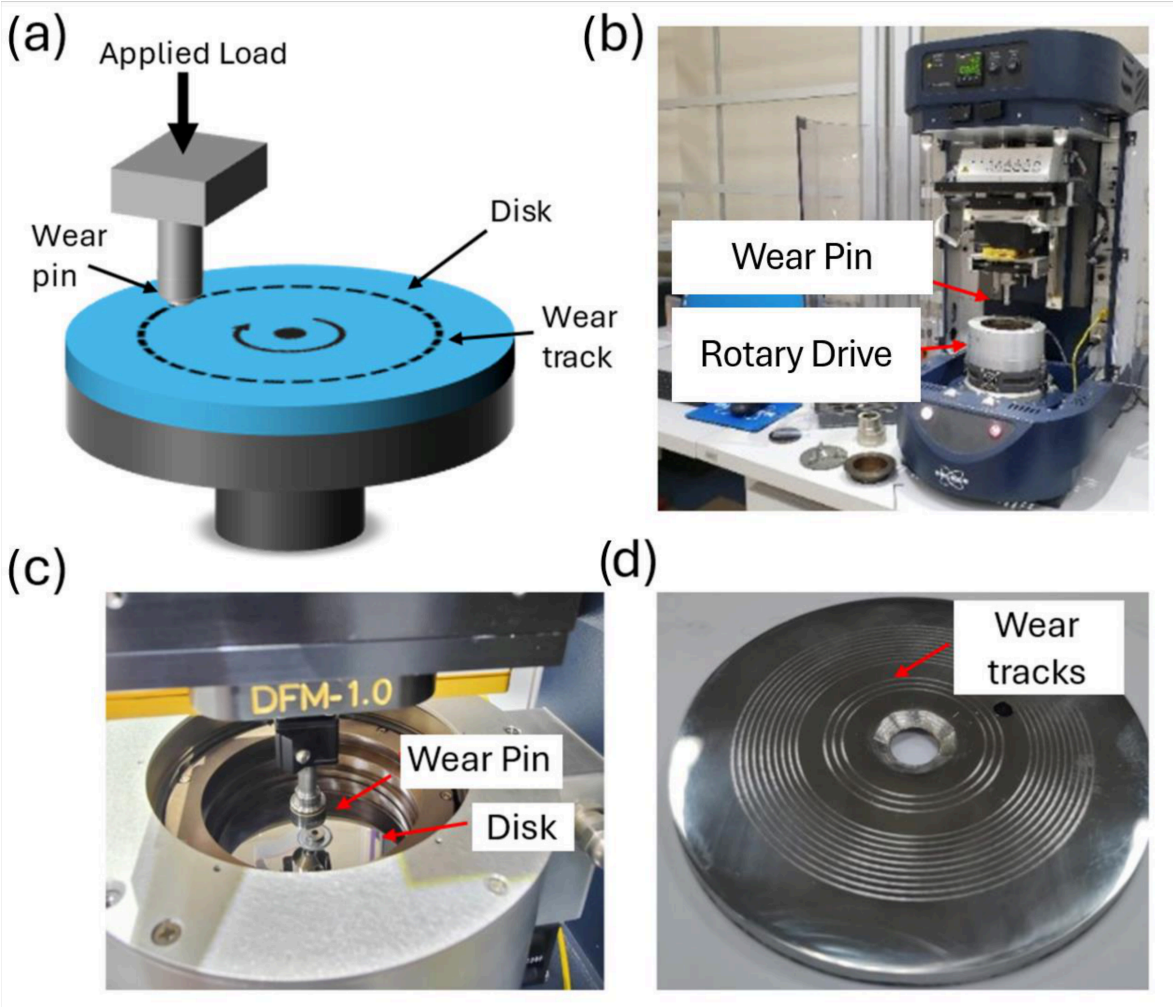
Pin-on-disk wear testing setup: (**a**) schematic representation of the pin-on-disk test; (**b**) testing machine Bruker UMT TriboLab; (**c**) wear pin maneuvering the disk surface before contact; (**d**) wear tracks on the disk surface post-wear test.

Bruker UMT Tribolab (Bruker Corporation, Billerica, MA, USA), a universal mechanical testing system, was used to perform pin-on-disk testing to measure wear following the procedures of ASTM G99 [37] Adjustments were made to enhance the testing performance based on the samples’ nature and prospective data. The sliding distance for all tests was set to $l_{s}$ = 100 m. Two distinct normal loads, $F_{N}$ = 1.5 N and $F_{N}$ = 2.5 N, were applied to investigate the effect of changing the normal load on wear behavior. The applied loads were carefully chosen to maintain the contact stress below the elastic modulus of the 316L stainless steel disks to avoid any elastic limits. Additionally, the effect of sliding speed was analyzed. The testing duration varied depending on the sliding speed to maintain a constant sliding distance for all tested samples. The pin-on-disk wear testing setup is shown in Figure 2.

The test specimen consisted of a 316L stainless steel disk with a diameter of 68 mm. The modulus of elasticity for the 316L is considered to be E = 200 GPa according to the EN 1993-1-4 standard [38] Previous investigations [39,40] on WAAM 316L stainless steel have shown that the experimentally measured Young’s modulus remains close to the conventional value for wrought austenitic stainless steel. However, small variations due to processing conditions and anisotropy have been reported. For the pin, a tungsten carbide ball with a diameter of 5 mm was selected, ensuring that it was thoroughly cleaned and free of contaminants before testing. The disk and ball properties are shown in Table 1. Surface roughness was measured using a TR1900 surface roughness tester (Beijing TIME High Technology Ltd., Beijing, China), with three roughness readings taken on each surface to confirm consistency and uniformity. The test materials were supplied with their respective material grades specified by the manufacturers. Accordingly, the chemical composition values listed in Table 1 were taken from the corresponding material specifications: DIN EN 10088-3:2024-04 [41] (Material No. 1.4404) for the stainless steel 316L disk and DIN 5401:2002 Bearing balls of Grade G10 were selected in accordance with DIN 5401:2002 [42] for the tungsten carbide ball. The hardness and surface roughness values were measured before the pin-on-disk tests.

The experiment employed a Bruker UMT TriboLab equipped with a DFM-1.0 load cell as the loading system (Bruker Scientific LLC, Billerica, MA, USA). This load cell has a range of 0.10 to 10 N, a resolution of 0.5 mN, and a suspension mechanism to enhance stability during testing. The rotary drive in the setup features a broad range of operational speeds, from 0.1 to 5000 RPM, allowing flexibility in rotational control. The drive delivers a maximum torque of greater than 5 Nm at 100 RPM and 2.5 Nm at 5000 RPM, with a maximum load capacity of up to 2000 N, meeting the demands of high-load wear testing. The normal loads are 1.5 N and 2.5 N, based on Hertzian contact theory for a tungsten carbide ball (E ≈ 650 GPa, ν ≈ 0.22) against the 316L disk (E = 200 GPa, ν ≈ 0.30); the calculated initial maximum contact pressures were approximately 1.09 GPa and 1.29 GPa for normal loads of 1.5 N and 2.5 N, respectively.

Specimen preparation was critical for achieving accurate results. The surface of the 316 stainless steel disk was polished to attain a mirror finish with a surface roughness (Ra) of less than 0.8 µm. After polishing, the disk was cleaned ultrasonically in ethanol—a non-chlorinated, non-film-forming cleaning agent—for five minutes to remove residual particles. The specimen was then dried with a hot air gun to ensure it was spotless and dry. The tungsten carbide ball underwent similar cleaning, being wiped with ethanol to remove any surface contaminants.

The test parameters for the pin-on-disk wear testing experiment ensured precise and controlled conditions. The test conditions were categorized using an index system, where each designation represents a unique combination of applied force and sliding speed. The sliding speed was maintained between 0.015 and 0.228 m/s, with a sliding distance of 100 m. The test was conducted at 25 °C under standard laboratory conditions, without specific humidity regulation. Table 2 summarizes the test parameters, detailing the corresponding force, sliding speed, and wear track radius for each test condition to achieve consistent and accurate wear measurements. The sliding distance was set to a constant l_s = 100 m, and each test was performed twice.

**Figure 3:**
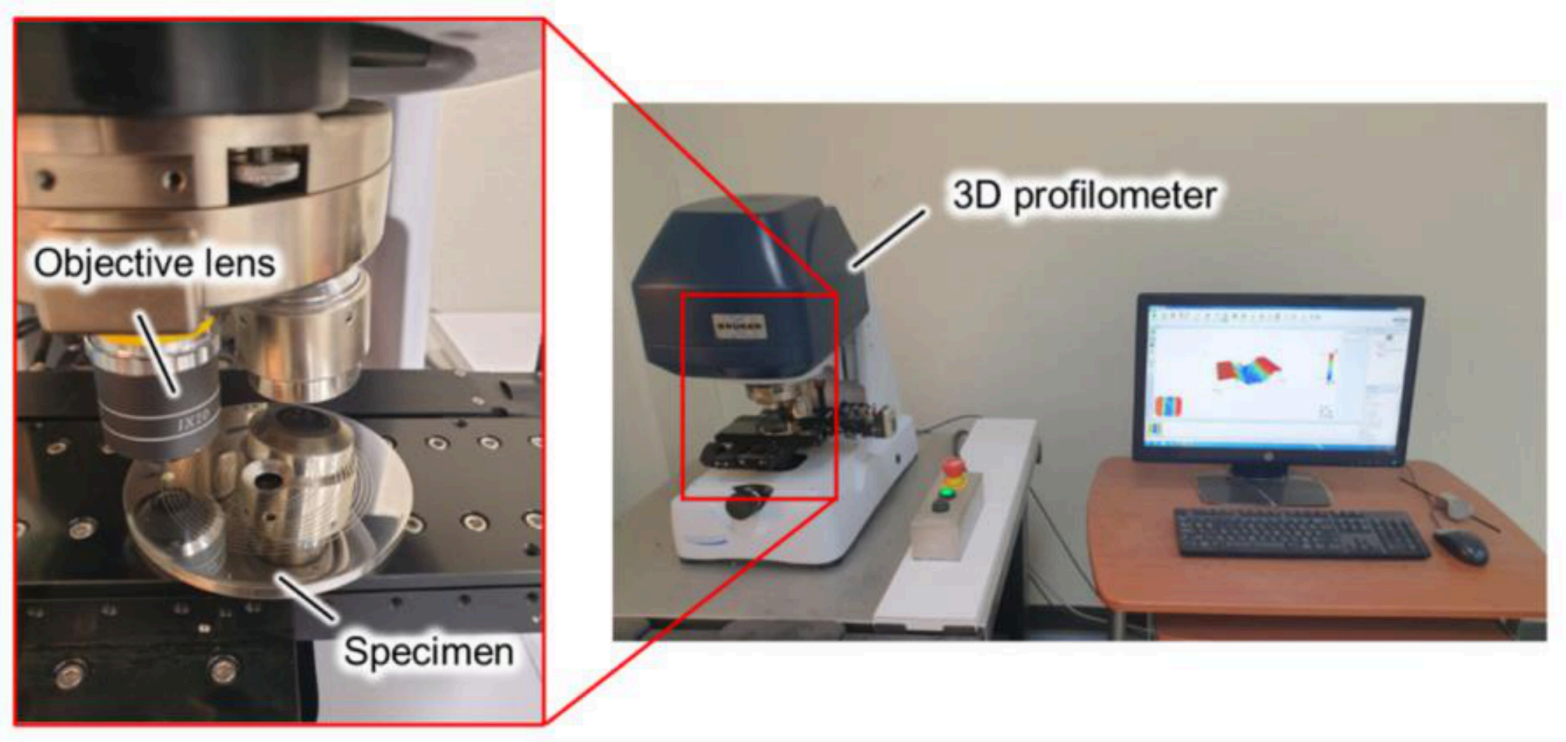
Wear profile measurement setup.

Post-test analysis involved several steps to assess the wear on the disk after the Pin-on-Disk wear testing experiment. The disk was first ultrasonically cleaned in ethanol, a non-chlorinated and non-film-forming cleaning agent, for five minutes, and then dried with a hot air gun. Wear measurements were then conducted using a Bruker ContourGT 3D optical profilometer (Bruker Corporation, Billerica, MA, USA) to capture two surface readings of each wear track. The setup for measuring the 3D wear profile is shown in Figure 3.

### 2.4. Hardness Testing Setup

The hardness testing was performed using a Zwick Roell Indentec hardness testing machine (ZwickRoell GmbH & Co. KG, Ulm, Germany). The printed plate by WAAM was cut into multiple samples, as illustrated in Figure 1c; a rectangular sample was reserved for hardness testing. The sample was cleaned and polished to ensure a smooth testing surface. Once prepared, the sample was placed in the testing machine, in which the force was set to 300 gf with a dwell time of 10 s. Hardness measurements were performed along the printing direction at 17 equally distributed locations. At each location, three indentations were made, and the hardness value was calculated as the average of these three measurements. This systematic approach improved the reliability of the measurements while enabling the evaluation of hardness variation along the printing direction.

## 3. Results

### 3.1. Microstructure Results and Discussion

The microstructural analysis reveals critical insights into phase characteristics and solidification processes involved in their formation during the WAAM process. Studying the distribution and morphology of phases such as delta-ferrite and gamma-austenite is crucial for investigating the mechanical properties and performance of the manufactured parts. The optical micrographs and solidification phenomena in the WAAM samples were analyzed, emphasizing the distinct regions and transformations that occur during manufacturing.

**Figure 4:**
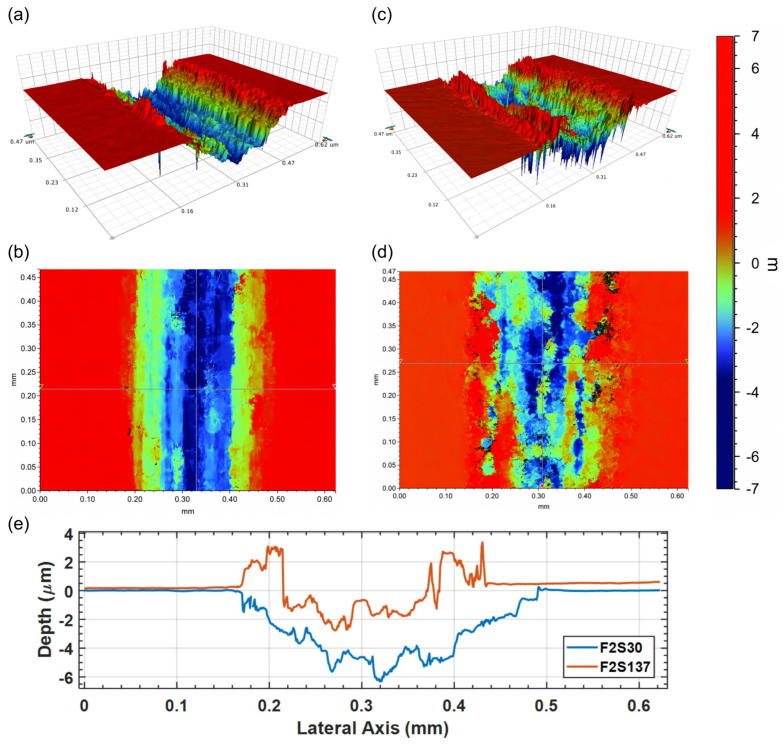
Wear profile measurements of two representative wear tracks (F2S30 and F2S137): (**a**) 3D wear track profile of F2S30; (**b**) 2D wear track profile of F2S30; (**c**) 3D wear track profile of F2S137; (**d**) 2D wear track profile of F2S137; (**e**) the depth variation along the lateral axis for both wear tracks.

Figure 4 presents the wear profile measurements of two representative tracks, F2S30 and F2S137, used to assess wear volume by first capturing their 3D profiles. The wear volume, or track depth, was measured based on profilometer data, and volume loss was calculated by converting linear measurements to wear volume, assuming negligible wear on the pin. The same procedure was applied to all wear tracks to ensure a consistent assessment of wear volume. Finally, the wear factor was determined using the following equation:

(1) $$k_{disk}=\frac{W_{v,disk}}{l_{s}\times F_{N}}$$

where $k_{disk}$ is the wear factor in mm^3^/N·m, $W_{v,disk}$ is the wear volume in mm^3^, $l_{s}$ is the total test sliding distance in m, and $F_{N}$ is the applied normal load in N.

**Figure 5:**
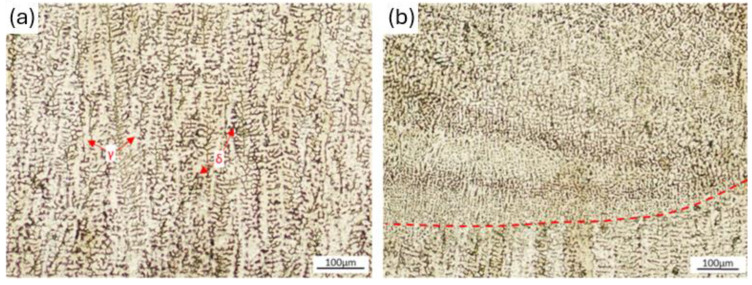
The microstructure at the central region of the longitudinal cross-section: (**a**) delta-ferrite in the gamma-austenite matrix; (**b**) coarser grains with vertical growth and residual ferrite in the middle zone.

Figure 5 shows optical micrographs of the WAAM sample, highlighting the delta-ferrite phase, visible in black, interspersed within the gamma-austenite matrix shown in Figure 5a. The microstructure depicted in Figure 5b represents the middle zone of the WAAM-built sample, which exhibits a different structure compared to the bottom zone. In the middle zone, the layers are deposited on previously solidified layers at elevated temperatures rather than at room temperature. As a result, the temperature gradient diminishes, leading to a decrease in cooling rates. This slower cooling promotes the formation of relatively coarser grains with vertical growth, accompanied by residual ferrite.

During the initial stages of solidification, delta-ferrite can form as the primary phase. Based on the established solidification behavior of austenitic stainless steels, ferrite-stabilizing elements such as Cr and Mo tend to partition preferentially toward delta-ferrite. In contrast, Ni favors the formation of gamma-austenite [20,43]. This elemental partitioning was not directly measured in the present study; therefore, this discussion is used only to interpret the observed ferrite–austenite morphology based on the reported literature.

The presence of residual delta-ferrite within the austenitic microstructure is consistent with previous observations for arc-additively manufactured 316L stainless steel. Chen et al. reported retained delta-ferrite within vertically oriented austenitic dendrites and showed that its morphology can change under the repeated thermal cycles generated by subsequent layer deposition [44]. Similarly, Wang et al. observed local variations in ferrite content within the multilayered structure of CMT-WAAM 316L [45]. Based on these previous findings, the lathy and skeletal delta-ferrite observed in Figure 5 is considered to be associated with the non-equilibrium solidification conditions and repeated thermal cycling inherent to the WAAM process. The partial transformation of primary delta-ferrite to gamma-austenite during solidification and subsequent thermal exposure has also been reported for austenitic stainless steels [43]. Therefore, the residual ferrite morphology observed in the present sample is interpreted based on these established solidification and thermal cycle effects.

Overall, Figure 5 highlights a ferrite–austenite solidification structure, in which gamma-austenite is the predominant phase and residual delta-ferrite is distributed within the austenitic matrix with lathy and skeletal morphologies. This general microstructural feature is consistent with previous observations for arc-additively manufactured austenitic stainless steels, where an austenitic matrix containing residual δ-ferrite has been reported [17]. The coarse and vertically developed grain structure observed in the middle region is also consistent with the directional heat flow associated with layer-by-layer arc deposition. A notable feature in the present sample is the coexistence of lathy and skeletal residual ferrite together with the coarser vertical grain growth in the middle zone, which may reflect the local thermal history and repeated thermal cycling during WAAM. Regarding defects, no obvious cracks or large pores were observed in the examined optical micrographs. However, the present optical examination was not intended as a quantitative defect assessment, and therefore the absence of smaller internal defects cannot be confirmed.

### 3.2. Wear Test Results

Wear tests were conducted on steel 316 under two loads, $F_{N}$ = 1.5 N and $F_{N}$ = 2.5 N, and the range of sliding speed from ν = 30 mm/s to ν = 229 mm/s will be presented and analyzed in this section. Material performance and wear resistance will be evaluated by measuring the coefficient of friction (COF) and friction force under dynamic conditions. Duplicate tests were performed for each condition to assess reproducibility. While dry sliding wear is inherently stochastic, the replicate tests exhibited consistent quantitative trends in wear volume and friction coefficient, and friction force, COF, and sliding time data were recorded and processed through a series of steps to remove any incomplete or anomalous readings. The sliding time for all tests was normalized to provide a consistent comparison basis. Also, a lowpass filter with a cutoff frequency of 0.1 Hz was applied to reduce noise and highlight key trends. Lastly, statistical analysis was performed for each test condition.

**Figure 6:**
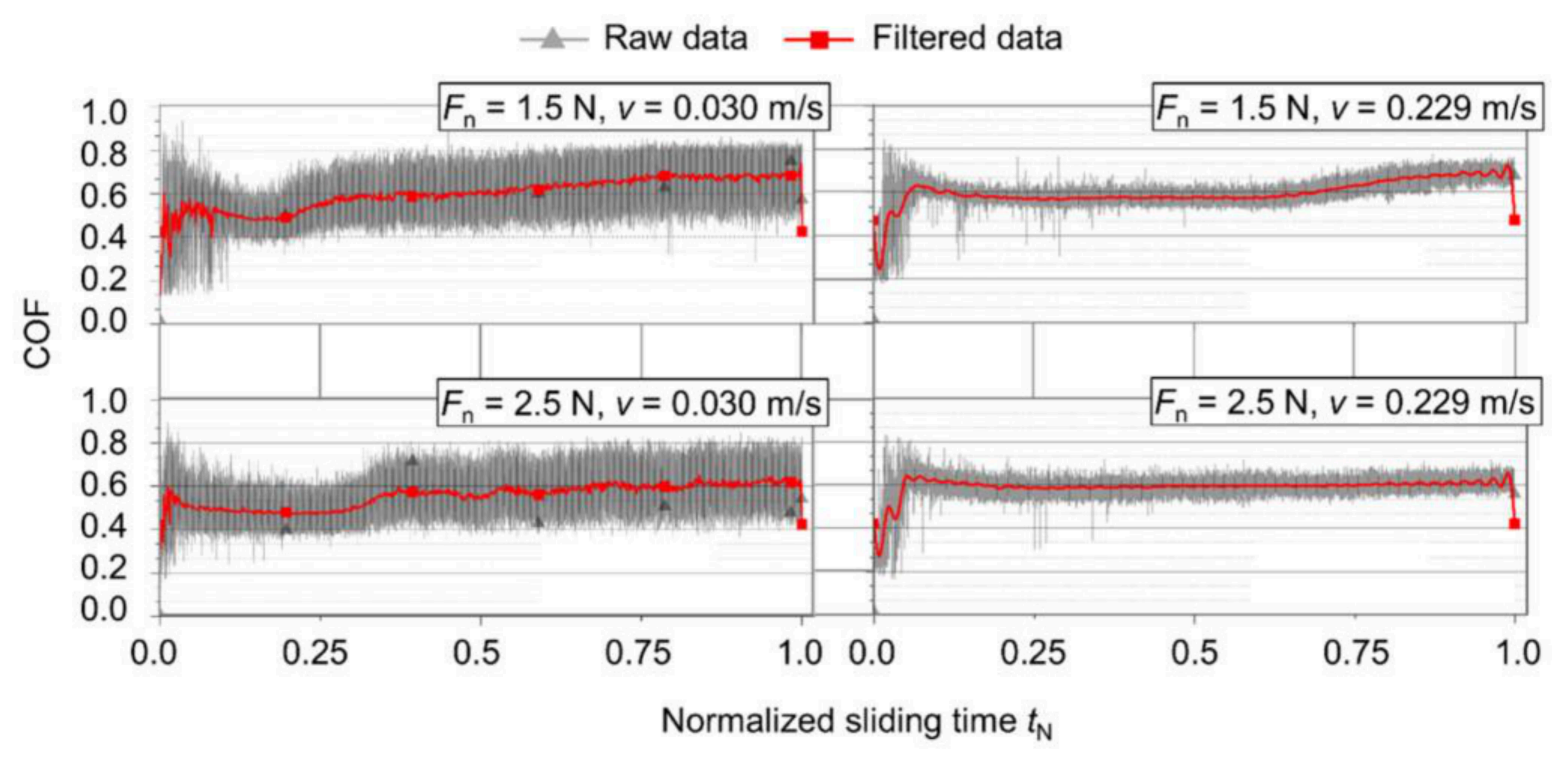
Time-resolved coefficient of friction data.

Figure 6 illustrates the time-resolved COF data collected for two representative normal forces, $F_{N}$ = 1.5 N and $F_{N}$ = 2.5 N, and two sliding speeds: ν = 0.03 m/s and ν = 0.229 m/s. Both raw and filtered COF data are presented to provide insights into dynamic behavior and underlying trends.

For COF, when the normal force is increased from 1.5 N to 2.5 N, average COF values demonstrate a slight variation, suggesting that the COF is relatively insensitive to the applied load in this range. Similarly, the average COF values did not increase when the sliding speed increased from ν = 0.03 m/s to ν = 0.229 m/s. The stability in the average COF across previous conditions highlights the consistent wear performance of the material under these test parameters.

The COF data exhibit dynamic behavior in all cases, with fluctuations observed around the mean value. At the lower sliding speed of ν = 0.03 m/s, the amplitude of fluctuations is more significant compared to the higher sliding speed of ν = 0.229 m/s. This indicates surface interaction variability due to more dominant adhesive wear mechanisms at reduced speeds. The WAAM process’s build direction also influences the dynamic signal, as the material layers are deposited in a specific orientation. Due to the material’s grain structure, this orientation could affect frictional behavior. However, further investigation is necessary to confirm this hypothesis and fully understand its contribution to the observed fluctuations.

**Figure 7:**
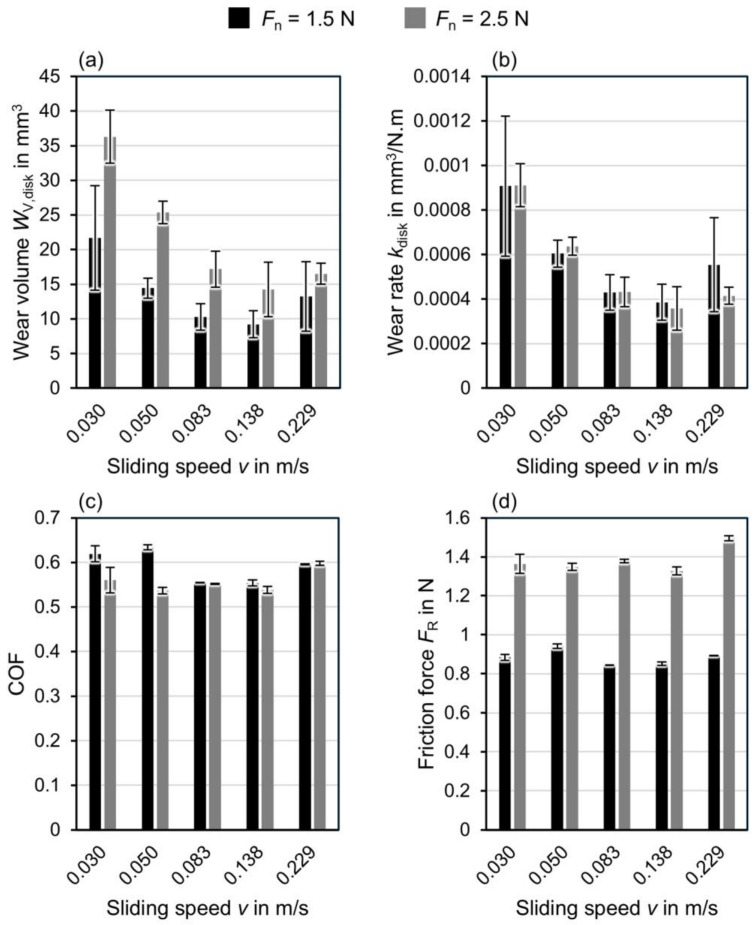
Wear test results with mean values plotted against sliding speed: (**a**) wear volume; (**b**) wear rate; (**c**) coefficient of friction; (**d**) friction force.

Figure 7 summarizes the mean values of the wear volume, wear rate, coefficient of friction (COF), and friction force obtained under different sliding speeds and applied normal loads. Figure 7a shows that the wear volume generally decreases with increasing sliding speed for both applied normal loads. As expected, the wear volume is consistently higher under the applied normal load of 2.5 N because of the greater contact force. A similar reduction in wear with increasing sliding speed has previously been reported for unlubricated 316 stainless steel under dry sliding conditions [46]. Figure 7b presents the wear factor *k*_disk_ as a function of sliding speed. The wear rate follows a trend similar to that of the wear volume, generally decreasing as the sliding speed increases. In contrast, the influence of the applied normal load on the wear rate is relatively small, indicating that, within the investigated test conditions, sliding speed has a greater influence on the specific wear performance than the applied load. Figure 7c illustrates the coefficient of friction (COF). The COF remains relatively stable, ranging from approximately 0.53 to 0.63 over the investigated sliding speeds, with only minor differences between the two applied normal loads. This indicates that the frictional response is only weakly influenced by sliding speed and normal load within the investigated parameter range. Figure 7d shows the friction force, which is consistently higher under the applied normal load of 2.5 N. This behavior is expected because the friction force is directly proportional to the applied normal load, while the relatively constant COF results in only small variations in friction force with sliding speed.

Overall, the measured wear factors and COF are in good agreement with values reported for conventionally manufactured 316 stainless steel under dry sliding conditions. Smith et al. [46] likewise observed that increasing sliding speed reduced the wear factor of unlubricated 316 stainless steel under low-load conditions, which is consistent with the trend observed in the present work. Furthermore, Tanwar and Jhavar [31] reported a wear factor of approximately 6.6 × 10^−6^ mm^3^/N·m and a COF of 0.7 for conventionally manufactured stainless steel. In contrast, additively manufactured 316L stainless steel exhibited lower wear factors of 2.3–6.4 × 10^−6^ mm^3^/N·m together with a reduced COF of approximately 0.5. These improvements were attributed to the refined microstructure and enhanced mechanical properties associated with the additive manufacturing process [31]. Similarly, Hsu et al. [47] demonstrated that the friction and wear behavior of austenitic stainless steels are strongly influenced by microstructural evolution during sliding, while Joshi et al. [48] reported that surface mechanical attrition treatment significantly improves the wear resistance of AISI 316L through surface hardening. Furthermore, Li et al. [49] showed that reinforcing SLM-fabricated 316L stainless steel with TiC particles further improved its tribological performance by reducing wear and friction compared with unreinforced 316L, highlighting the important role of microstructural modification in enhancing wear resistance.

### 3.3. Hardness Test Results

**Figure 8:**
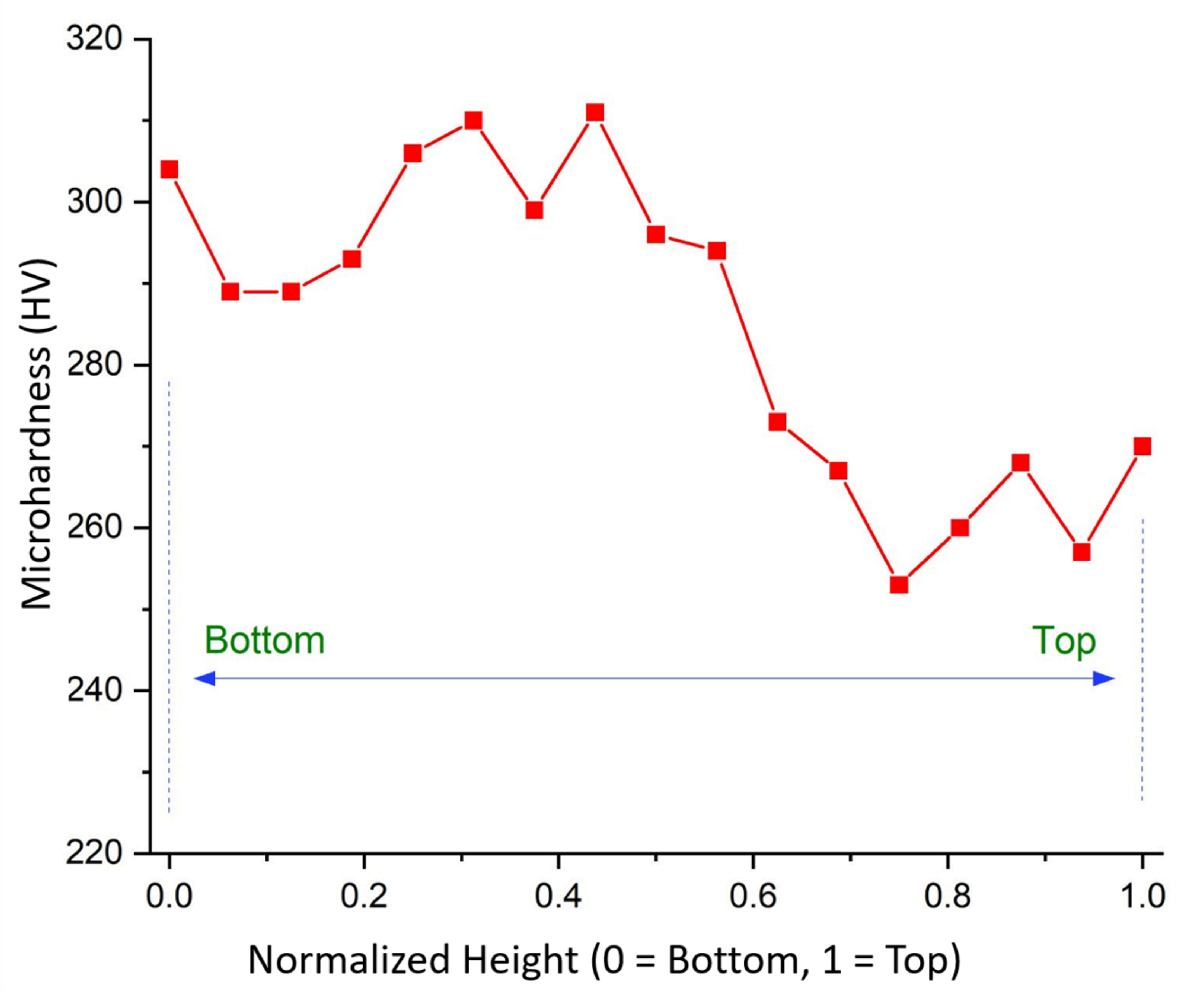
Microhardness of the printed samples along the printing direction.

Figure 8 illustrates the hardness values of the WAAM-printed samples, showcasing the results of multiple indentations conducted along the z-direction. The vertical axis shows the microhardness values, and the horizontal axis represents the normalized position along the specimen height, where 0 corresponds to the bottom of the build, and 1 represents the top of the WAAM sample. This investigated the influence of the printing direction on the hardness of the printed sample. Each layer has a unique thermal cycle, resulting in distinct thermal histories that affect the mechanical properties of the printed sample [50]. The results showed that the hardness values fluctuated from 253 HV to 311 HV. Given that the tested sample has a height of 20 mm and a layer thickness of approximately 3 mm, the influence of different thermal histories was not evident in the hardness measurements.

## 4. Conclusions

This study analyzed the wear and tribological behavior of WAAM-fabricated austenitic stainless steel 316L under dry sliding conditions. The results highlight several important trends.

- WAAM-produced 316L exhibited a stable coefficient of friction of approximately 0.6 over the investigated loading conditions.
- Wear volume generally decreased as sliding speed increased, whereas lower normal loads resulted in lower material loss.
- The wear factor showed limited dependence on the applied normal load.
- Optical microscopy revealed a ferrite–austenite microstructure containing residual δ-ferrite, which is consistent with the stable tribological response observed.
- Microhardness remained between approximately 253 and 311 HV, indicating relatively uniform mechanical properties along the build direction.

## Figures and Tables

**Table 1:** Properties of test specimen in pin-on-disk test.

Test Specimen/ Material	Ball/Tungsten Carbide	Disk/Stainless Steel 316L (X2CrNiMo17-12-2) ^a^
Chemical composition mass fraction in %	WC: 94 Co: 6	C: 0.030, Si: 1.00, Mn: 2.00, P: 0.045, S: 0.030, Cr: 16.5 to 18.5, Mo: 2.00 to 2.50, Ni: 10.0 to 13.0, N: 0.10
Hardness	(90 to 91.5) HRA (1400 to 1550) HV	(253 to 311) HV
Mean surface roughness Rz [µm]	--	0.022
Mean surface roughness Ra [µm]	0.02 ^b^	0.142

^a^ According to DIN EN 10088-3 material number 1.4404. ^b^ According to DIN 5401:2002 (G10).

**Table 2:** Pin-on-disk testing parameters.

Index	Normal Force	Sliding Speed	Wear Track Radius
	[N]	[mm/s]	[mm]
F1S15	1.50	15.00	11.00
F2S15	2.50	15.00	13.50
F1S30	1.50	30.00	11.00
F2S30	2.50	30.00	18.75
F1S50	1.50	50.00	12.25
F2S50	2.50	50.00	22.50
F1S83	1.50	83.00	17.50
F2S83	2.50	83.00	25.00
F1S137	1.50	137.78	19.50
F2S137	2.50	137.78	21.50
F1S228	1.50	228.71	23.50
F2S228	2.50	228.71	25.50

## Data Availability

The data presented in this study are available from the corresponding author upon reasonable request. The data are not publicly available because they form part of an ongoing research project and include raw experimental data that are not currently maintained in a public repository.
